# Distinct Transmissibility Features of TSE Sources Derived from Ruminant Prion Diseases by the Oral Route in a Transgenic Mouse Model (TgOvPrP4) Overexpressing the Ovine Prion Protein

**DOI:** 10.1371/journal.pone.0096215

**Published:** 2014-05-05

**Authors:** Jean-Noël Arsac, Thierry Baron

**Affiliations:** Agence nationale de sécurité sanitaire de l'alimentation, de l'environnement et du travail (Anses), Unité Maladies Neuro-dégénératives, Lyon, France; Deutsches Zentrum für Neurodegenerative Erkrankungen e.V., Germany

## Abstract

Transmissible spongiform encephalopathies (TSEs) are a group of fatal neurodegenerative diseases associated with a misfolded form of host-encoded prion protein (PrP). Some of them, such as classical bovine spongiform encephalopathy in cattle (BSE), transmissible mink encephalopathy (TME), kuru and variant Creutzfeldt–Jakob disease in humans, are acquired by the oral route exposure to infected tissues. We investigated the possible transmission by the oral route of a panel of strains derived from ruminant prion diseases in a transgenic mouse model (TgOvPrP4) overexpressing the ovine prion protein (A_136_R_154_Q_171_) under the control of the neuron-specific enolase promoter. Sources derived from Nor98, CH1641 or 87V scrapie sources, as well as sources derived from L-type BSE or cattle-passaged TME, failed to transmit by the oral route, whereas those derived from classical BSE and classical scrapie were successfully transmitted. Apart from a possible effect of passage history of the TSE agent in the inocula, this implied the occurrence of subtle molecular changes in the protease-resistant prion protein (PrPres) following oral transmission that can raises concerns about our ability to correctly identify sheep that might be orally infected by the BSE agent in the field. Our results provide proof of principle that transgenic mouse models can be used to examine the transmissibility of TSE agents by the oral route, providing novel insights regarding the pathogenesis of prion diseases.

## Introduction

Transmissible spongiform encephalopathies (TSEs) are neurodegenerative diseases that affect both humans and animals. Typical features include characteristic spongiform changes in the brain associated with neuron loss, an absence of inflammatory response, and accumulation in the brain, and sometimes in lymphoid tissues, of an abnormal, partially protease-resistant (PrPres) form of the neuronal prion protein (PrP) encoded by the *prnp* gene of the host [1]. This disease-associated protein is associated with transmissibility of the disease.

The routes by which TSEs occur under natural conditions, have not been fully established. However, scrapie and chronic wasting disease (CWD), in small ruminants and cervids respectively, show horizontal transmissibility under natural conditions [2], [3], and many prion diseases are acquired by oral exposure, e.g., bovine spongiform encephalopathy (BSE) [4], transmissible mink encephalopathy (TME) [5], kuru and, most probably, variant Creutzfeldt–Jakob disease (vCJD) in humans [6]. As a result of increased surveillance in the past years, atypical and/or rare TSEs have been identified in cattle (H-type and L-type BSEs) and in small ruminants (Nor98 and scrapie isolates reminiscent of CH1641 experimental scrapie) [7], [8], [9], [10]. The origins of these TSEs remain debated but at least some of them (H-type and L-type BSEs, Nor98) probably arise sporadically [10], [11], such as most cases of Creutzfeldt-Jakob disease (CJD) in humans [12], although particular sequences of the *prnp* gene (A_136_H_154_Q_171_ and A_136_F_141_R_154_Q_171_ genotypes) have a major predisposing influence in the case of No98 [13].

Prion diseases pathogenesis has been shown to be heavily dependent on complex interactions between factors specific to the strain of transmissible agent, the route and dose of exposure and the host [13], [14]. In this context, experimental transmissions of different TSEs provide useful information about the potential risk of transmission and for understanding disease pathogenesis. We therefore decided to investigate the possible transmissibility by the oral route of a large panel of TSEs derived from prion diseases of ruminants in a transgenic mouse model (TgOvPrP4), after two passages of these sources by the intra-cerebral route [15], [16], [17], [18], [19], [20]. This choice was made to avoid inoculations of tissues derived from different species (cattle or sheep) with different PrP primary sequences.

In this study our objectives continued our efforts to examine in details the pathogenesis of prion diseases in the TgOvPrP4 transgenic mouse model expressing PrP under the control of a neuron-specific enolase promoter. Our previous studies indeed already showed that, although we failed to detect ovine PrP outside the central nervous system, TgOvPrP4 mice could be successfully infected by intra-peritoneal route with a classical scrapie source [21] and showed PrPres accumulation into lymphoid tissues with some prion strains, including classical scrapie and BSE [21], [22].

## Materials and Methods

### Ethics statement

All mouse transmission experiments were performed in the biohazard prevention area (A3) of the ANSES-Lyon animal facilities, in accordance with the guidelines of the French Ethics Committee (decree 87–848) and European Community Directive 86/609/EEC, with the relevant approval for licensed individuals (LL 69 387 191) to carry out animal experiments (A 69 387 0801) according to a protocol approved (Permit n°98) by the Committee on the Ethics of Animal Experiments (CREEA of the Région Rhône Alpes Auvergne).

### TSE sources

All samples used for the 3^rd^ passage (oral and intra-cerebral challenge of TgOvPrP4 mice) consisted of ovine transgenic (TgOvPrP4) mouse brains of the different BSE or scrapie sources obtained after two successive passages by intra-cerebral route.

Inocula were derived from i) natural cases of classical BSE in cattle and classical scrapie in sheep [23], ii) atypical natural TSE isolates collected by active surveillance of scrapie (“CH1641 like”, Nor98) [16], [24] or of BSE (L-type BSE) [18], iii) experimental samples including classical BSE and CH1641 in AA_136_RR_154_QQ_171_ sheep [16], [25], transmissible mink encephalopathy (TME) in cattle [18] or scrapie strains passaged in wild-type mice (87V, C506M3) [17].

### Transmission studies in mice

The TgOvPrP4 transgenic mice used in the present study express the ovine PrP protein (A_136_R_154_Q_171_ genotype) under the control of the neurone-specific enolase promoter, and do not express the murine PrP protein [15].

For intra-cerebral inoculations, experimental groups (n = 6 to 12) of four to six week old female TgOvPrP4 mice were anaesthetized (80 µl of 0.8% ketamin–0.12% xylazine) then inoculated intracerebrally (20 µl per mouse) with a 1% solution (wt/vol) in glucose (5%) of homogenates prepared from brain samples of mice at the terminal stage of the disease during a second passage of the different TSE sources, as previously described [15], [16], [17], [18], [19], [20], [23], [25].

Transmissions by the oral route were carried out with experimental groups (n = 6) of four to six week old female TgOvPrP4 mice challenged with 10% brain homogenates in glucose 5% (wt/vol) from intra-cerebrally challenged TgOvPrP4 mice at the terminal stage of the disease during a second passage of the different TSE sources. For intra-gastric administration, mice were housed for 2 hours in bedding- and food-free cages then individually fed (100 µl per mouse) *via* a flexible polypropylene catheter inserted over the tongue about 1 to 2 cm into the oesophagus.

Mice were supplied with food and drink *ad libitum*, then checked at least twice weekly for the presence of clinical signs indicative of TSE. When signs occurred, the mice were monitored daily and were sacrificed with an overdose of anaesthetic solution (200 µl of 0.8% ketamine–0.12% xylazine) if they exhibited any signs of distress or confirmed evolution of clinical signs of prion disease. A few animals were found dead. The whole brain and the spleen from each mouse available for appropriate sampling was frozen and stored at −80°C until Western blot analysis for PrPres detection.

### Western blot analysis of PrPres

Details of the Western blot analyses have been provided in previous publications describing transmission of the TSE sources used in this study. Briefly, brain and spleen from TgOvPrP4 mice inoculated with strain derived from Nor98 scrapie isolate were examined by TeSeE WB (Bio-Rad) following the manufacturer's recommendations, as previously described [19], [24]. For other TSE sources, Western blot were performed after PrPres extraction from brain or spleen by ultra-centrifugation, as described [22]. In some experiments, deglycosylation was performed using PNGase F as previously described [16].

PrPres was detected by SHa31 or SAF84 antibodies (4 µg/ml in PBST), recognizing the 148-YEDRYYRE-155 or 167-RPVDQY-172 ovine PrP sequence respectively, and peroxidase-labelled conjugate (Cliniscience) against mouse IgG (1∶2,500 in PBST). Bound antibodies were detected by enhanced chemiluminescence (ECL, Amersham). PrPres signals were visualized either on film (Biomax; Kodak) or directly in an image-analysis system (Versadoc; Bio-Rad) and Quantity One software (Bio-Rad).

## Results

### Transmissibility of TSE sources by the oral route in TgOvPrP4 mice

**Figure 1:**
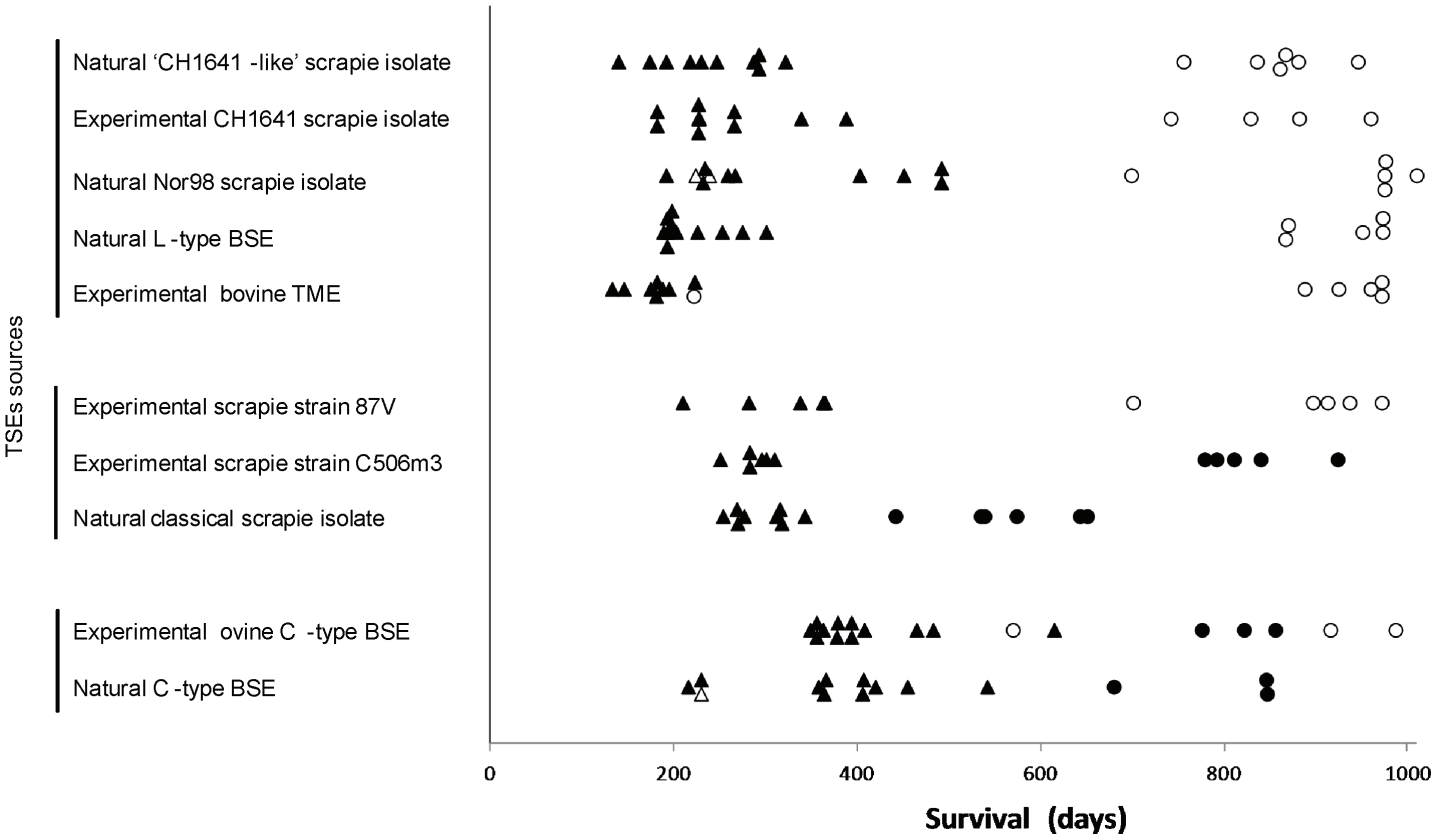
Survival periods and PrPres detection of individual TgOvPrP4 mice challenged by oral *versus* intra-cerebral route. ▵: intra-cerebral, O: oral. Empty circles or triangles correspond to PrPres negative mice whereas full circles or full triangles represent PrPres positive mice.

We examined the transmissibility by the oral route of ten TSE sources from cattle or sheep previously described in the TgOvPrP4 transgenic mouse model, after two serial passages by the intra-cerebral route in this mouse line. The results are summarized in Tables 1 and S1, which show (i) the survival periods of the mice (ii) PrPres detection by Western blot in brains and spleens and (iii) clinical signs of TSE, in comparison to a second and third passage by the intra-cerebral route. Survival periods and PrPres detection in each individual mouse in these experiments are shown in Figure 1.

**Table 1:** TSE transmission results by the oral *versus* the intra-cerebral route in TgOvPrP4 ovine transgenic mice.

TSE sources	Survival (analyzed/inoculated)						Brain PrPres			Spleen PrPres		
	2^nd^ i.c.		3^rd^ i.c.		3^rd^ oral		2^nd^ i.c.	3^rd^ i.c.	3^rd^ oral	2^nd^ i.c.	3^rd^ i.c.	3^rd^ oral
Natural ‘CH1641-like’ scrapie isolate	232±38	(11/12)	253±66	(10/11)	858±62	(6/6)	**11/11**	**10/10**	0/6	0/5	0/10	0/6
Experimental CH1641 scrapie isolate	220±31	(11/12)	244±58	(11/12)	853±92	(4/6)	**11/11**	**11/11**	0/4	0/5	0/9	0/4
Natural Nor98 scrapie isolate	317±117	(11/12)	ND		927±128	(5/6)	**9/11**	ND	0/5	0/11	ND	0/5
Natural L-type BSE	202±26	(9/11)	220±39	(11/12)	935±52	(6/6)	**9/9**	**11/11**	0/6	**3/5**	**9/9**	0/6
Experimental Bovine TME	234±27	(9/11)	178±28	(8/11)	823±296	(6/6)	**3/9**	**8/8**	0/6	**1/3**	**4/5**	0/6
Experimental scrapie strain 87V	270±55	(10/11)	312±66	(5/6)	884±106	(5/6)	**10/10**	**5/5**	0/5	0/7	0/5	0/5
Experimental scrapie strain C506m3	333±26	(12/12)	287±21	(6/6)	829±58	(5/6)	**12/12**	**6/6**	**5/5**	**6/6**	**6/6**	**5/5**
Natural classical scrapie isolate	295±31	(8/11)	ND		564±78	(6/6)	**8/8**	ND	**6/6**	**7/7**	ND	**5/5**
Experimental Ovine C-type BSE	356±62	(12/12)	412±12	(12/12)	821±143	(6/6)	**12/12**	**12/12**	**3/6**	**5/5**	**6/6**	**1/5**
Natural C-type BSE	354±48	(10/11)	415±61	(8/11)	791±96	(3/6)	**10/10**	**8/8**	**3/3**	**5/5**	**8/8**	**3/3**

Survival periods (means +/− standard deviations) and the number of analyzed mice for the ten TSE sources are shown together with the results of PrPres detection in brain and spleen (number of PrPres positive mice/number of mice examined). The results obtained for the second intra-cerebral passage (2^nd^ i.c.) are shown in comparison with those obtained for the third passage by the oral route (3^rd^ oral) or the intra-cerebral route (3^rd^ i.c.). ND: no data.

Regarding clinical signs (Table S1), mice inoculated by oral route generally appeared thin, as they were generally sacrificed or found dead at late ages. Paresis was observed with two classical scrapie sources (a French natural scrapie case or the mouse-adapted C506M3 experimental strain) after oral challenge, whereas this clinical signs is generally observed in mice inoculated by the intra-cerebral route (2^nd^ and 3^rd^ passage) for all the 10 TSE sources. Prostration, another criteria generally observed in mice inoculated by the intra-cerebral route, was observed with the three classical scrapie sources (a French natural scrapie case, the mouse-adapted C506M3 experimental strain or the mouse-adapted 87V experimental strain) but also with the sources derived from experimental bovine TME and the natural Nor98 scrapie isolate. Additionally, clinical signs as dorsal kyphosis, plastic tail and foot clasping were included as signs participating to the definition of the clinical endpoint.

Considering the detection of PrPres in both brains and spleens of the mice (Table 1), the TSE sources derived from classical BSE sources (cattle or ovine BSE) and from two classical scrapie sources (a French natural scrapie case or the mouse-adapted C506M3 experimental strain) were successfully transmitted by the oral route. However, the mean survival periods exceeded 2 years, except in mice that received the source derived from natural scrapie source (mean 564 days post-inoculation (d.p.i.)).

On the other hand, we failed to transmit the six other TSE sources, notably the sources derived from CH1641 and Nor98 scrapie, L-type BSE and bovine-passaged transmissible mink encephalopathy (cattle-passaged TME). Interestingly, only two of these TSE sources, derived from cattle (L-BSE and cattle-passaged TME), appeared to be lymphotropic in TgOvPrP4 mice after intra-cerebral inoculation, which was not the case for the four sources derived from scrapie (Nor98, “CH1641-like” natural scrapie, experimental sources CH1641 or 87V).

### Changes of PrPres cleavage in the brains of TgOvPrP4 mice orally challenged by BSE

**Figure 2:**
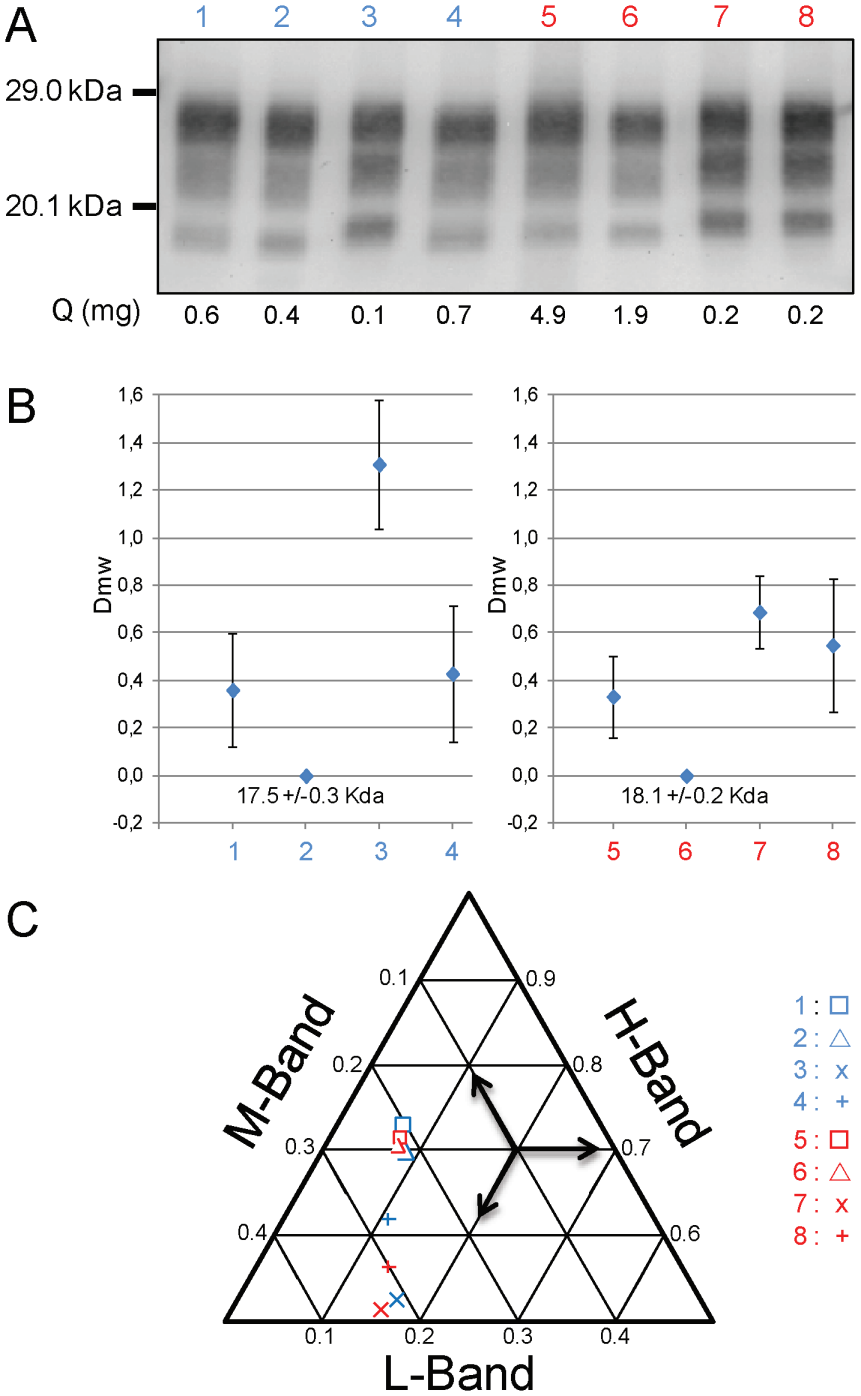
Western blot of brain PrPres in TgOvPrP4 mice infected with classical scrapie or BSE. Mice were challenged by oral (lane 5–8) or the intra-cerebral route (lanes 1–4). A) Western blot detection of PrPres by Sha 31 antibody of different sources derived from classical BSE in cattle (lanes 1, 5), classical BSE in sheep (lanes 2, 6), C506M3 scrapie strain (lanes 3, 7) and natural classical scrapie isolate (lanes 4, 8). The equivalent tissue quantities loaded per lane are indicated at the bottom of each lane. Bars to the left indicate the 29.0 and 20.1 kDa marker positions. B) Differences of the unglycosylated PrPres apparent molecular masses compared to that in mice infected with source derived from ovine BSE by the same inoculation route (means +/− SD of 5 repeated assays). The raw apparent molecular mass of unglycosylated PrPres is indicated for the ovine BSE infected mouse. C) Tern-plot representation of the proportions of diglycosylated (H), monoglycosylated (M) and unglycosylated (L) bands of PrPres for different sources derived from classical BSE in cattle (□), classical BSE in sheep (▵), C506M3 scrapie strain (X) and natural classical scrapie isolate (+) (blue: intra-cerebral, red: oral). The means for all repetitions (n = 5) are plotted.

The biochemical features of PrPres were then compared in the brains of mice inoculated by the oral or the intra-cerebral routes (Figure 2).

When sources derived from classical BSE and classical scrapie were compared, the characteristic high levels of diglycosylated PrPres (∼70%) were still found in mice orally-challenged with BSE, which were clearly distinct from mice orally challenged with classical scrapie sources (<60%)(Figure 2C).

**Figure 3:**
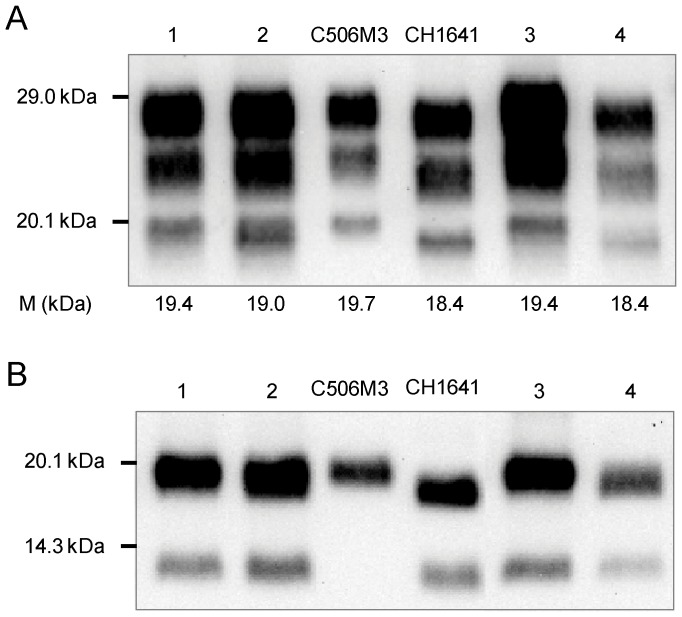
Western profiles in TgOvPrP4 mice infected with source derived from a natural scrapie isolate. PrPres detected, using SAF84 antibody, from four individual TgOvPrP4 mice infected with a natural scrapie isolate [15], at the second passage by the intra-cerebral route (lanes 1–4). PrPres of TgOvPrP4 mice infected with the C506M3 and CH1641 experimental scrapie sources were used as controls. A) The apparent molecular mass measured for the unglycosylated PrPres band in this representative Western blot is indicated at the bottom of each lane. Bars to the left indicate the 29.0 and 20.1 kDa marker positions. B) PrPres was analysed after PNGase deglycosylation. Bars to the left indicate the 20.1 and 14.3 kDa marker positions.

However molecular discrimination of sources derived from BSE and scrapie, based on measuring differences in the apparent molecular mass of the unglycosylated PrPres, was much more difficult. The apparent molecular mass of unglycosylated PrPres in source derived from BSE was clearly much higher in mice infected by the oral route, so that BSE was sometimes indistinguishable from scrapie for this criterion (Figure 2B, lanes 5 and 8). Whereas these differences were >1 kDa for the C506M3 strain inoculated by the intra-cerebral route (Figure 2B, lane 3), the apparent molecular mass for one mouse inoculated intra-cerebrally with a source derived from classical natural scrapie was low (Figure 2B, lane 4), similar to that found for the two BSE sources by this inoculation route (Figure 2B, lanes 1 and 2). This same mouse was also the only scrapie-infected mouse in which the proportion of diglycosylated PrPres was >60% (Figure 2C). However, other mice in this experimental group showed a classical scrapie pattern, with a higher apparent molecular mass for the unglycosylated PrPres band and, as in CH1641, C-terminally cleaved PrPres (∼14 kDa for the unglycosylated band) specifically recognised by a C-terminal antibody (SAF84) was detected in all mice (Figure 3).

### Changes of PrPres glycoform ratios in the spleen of TgOvPrP4 mice orally challenged by scrapie

**Figure 4:**
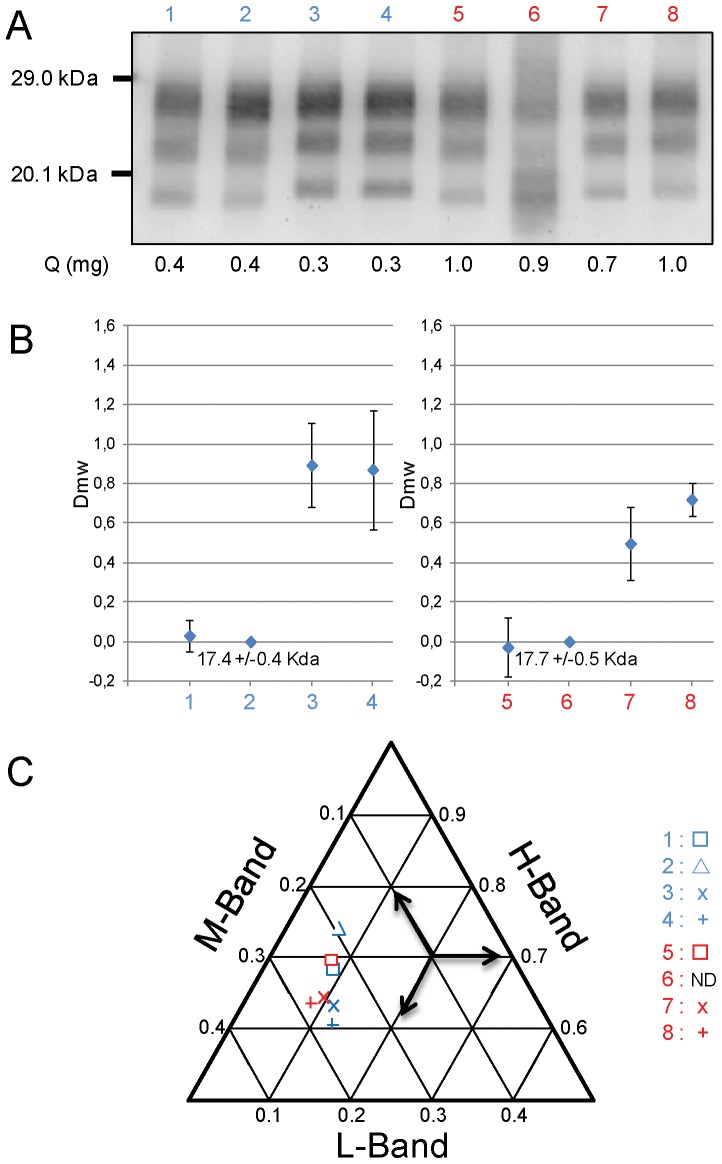
Western blot of spleen PrPres in TgOvPrP4 mice infected with classical scrapie or BSE. Mice were challenged by oral (lane 5–8) or the intra-cerebral route (lanes 1–4). A) Western blot detection of PrPres by Sha 31 antibody of different sources derived from classical BSE in cattle (lanes 1, 5), classical BSE in sheep (lanes 2, 6), C506M3 scrapie strain (lanes 3, 7) and natural classical scrapie isolate (lanes 4, 8). The equivalent tissue quantities loaded per lane are indicated at the bottom of each lane. Bars to the left indicate the 29.0 and 20.1 kDa marker positions. B) Differences of the unglycosylated PrPres apparent molecular masses compared to that in mice infected with source derived from ovine BSE by the same inoculation route (means +/− SD of 5 repeated assays). The raw apparent molecular mass of unglycosylated PrPres is indicated for the ovine BSE infected mouse. C) Tern-plot representation of the proportions of diglycosylated (H), monoglycosylated (M) and unglycosylated (L) bands of PrPres for different sources derived from classical BSE in cattle (□), classical BSE in sheep (▵), C506M3 scrapie strain (X) and natural classical scrapie isolate (+) (blue: intra-cerebral, red: oral). The means for all repetitions (n = 5) are plotted.

The biochemical features of PrPres in the spleens of mice inoculated by oral or intra-cerebral routes were similarly compared (Figure 4).

Concerning PrPres mobility (Figure 4B), it was again less easy to discriminate between sources derived from BSE and scrapie in orally-challenged mice, but to a lesser extent than in the brain since a 0.3–0.5 kDa increase in molecular mass was observed for the unglycosylated PrPres of source derived from BSE (compared to 0.7–0.9 kDa in the brain). As a result, BSE can still be readily discriminated from scrapie by applying this criterion. Interestingly, by intra-cerebral route, for the mouse which showed a PrPres of low apparent molecular mass in the brain after inoculation with a natural scrapie source, scrapie could be readily discriminated from BSE in the spleen, where the apparent molecular mass of PrPres was still high (Figure 4B, lane 4).

Examination of the PrPres glycoform ratios revealed an increased proportion of diglycosylated PrPres (approximately 61%–63%) after inoculation with scrapie by both routes (Figure 4C), as compared to the PrPres ratios found in the brain, so these glycoform proportions were closer to that of BSE.

## Discussion

Our data show that TSE agents inoculated by the oral route can be transmitted in a transgenic mouse model (TgOvPrP4) overexpressing the ovine PrP protein (A_136_ R_154_ Q_171_) on a murine *prnp*-/- background under the control of the neuron-specific enolase promoter [15]. This extends our previous demonstrations of the transmission of two classical scrapie isolates by intra-peritoneal route in this mouse model [21], which had been unexpected given our failure to detect ovine PrP expression outside the central nervous system. However, transmission of a scrapie source had also been reported in a similar transgenic mouse model expressing the hamster prion protein (NSE HaPrP/MoPrP-/-) and challenged by either the intra-peritoneal or the oral route [26]. This suggested that PrP expression in the peripheral nerves might be sufficient for infection of the brain. Transmission has now been observed with the TgOvPrP4 ovine transgenic model, not only for two sources derived from classical scrapie (a natural scrapie isolate or the C506M3 experimental strain) but also for two sources derived from classical BSE (from cattle or after experimental passage in sheep).

However, it must be emphasized that we did not use brain samples from ruminant isolates (cattle or sheep) but chose to carry out the study using brains from TgOvPrP4 mice intracerebrally inoculated with the prion diseases. We cannot fully exclude possible biological changes after serial intracerebral passages in the ovine transgenic mouse model, including regarding the possible transmission by the oral route. However, this allowed to examine the properties after the same passage history in a same experimental ovinised host and avoid to compare experiments from brain pieces derived from different species (cattle or sheep). Survival periods at primary passage from bovine tissues were already quite long by intra-cerebral from cattle brain tissues, especially for L-BSE (>600 d.p.i.). As a result, life expectancy of the mice could prevent in practice any possibility to identify any transmission of the disease by the oral route.

Nevertheless, the transmission of both sources derived from BSE and scrapie allowed examination of the PrPres molecular differences. For a given sources derived from BSE of either bovine or ovine origin, the apparent molecular mass for PrPres was consistently higher in orally challenged mice, as has already been reported following experimental transmission of BSE in sheep [27]. These slight differences in molecular mass were observed in the brain and to a lesser extent in the spleen. As a result, molecular discrimination between BSE and scrapie, especially in mouse brains, was less straightforward when based on PrPres analyses. This raises concern about our ability to correctly identify sheep that might be orally infected by the BSE agent in the field, from discriminatory analyses of PrPres in the brain [28], [29] Indeed the strategy currently used for the discrimination of scrapie and BSE in small ruminants in Europe, is essentially based on the Western blot analyses of the PrPres cleavage in the brain [30].

PrPres molecular changes could suggest a possible emergence of a new strain, although it is also possible that striking molecular changes occur without any detectable strain alteration, as illustrated for instance recently by major changes of glycosylation patterns in BSE transmitted in a porcine transgenic mouse model [31]. As this was revealed by further passage in bovine transgenic mice in this last study, strain typing from our orally infected mice showing altered PrPres features will require further passage by intracerebral route in an appropriate experimental model as it has been well established that the route of infection can strongly influence the apparent phenotype of a TSE strain [32], [33].

Examination of the molecular features of the PrPres derived from scrapie revealed a modification of the biochemical profile with a PrPres apparent molecular mass and a high proportion of diglycosylated PrPres (>60%) similar to that of BSE-infected mice, but this was the case of a single mouse among those intra-cerebrally inoculated with the strain derived from natural scrapie. This is reminiscent of the emergence of scrapie with CH1641 features that was described after intra-species experimental transmission in sheep [34]. This is another circumstance when molecular discrimination between scrapie and BSE is more difficult [7], [28]. However, in contrast to sources derived from BSE, no significant molecular differences were otherwise observed between mice orally or intra-cerebrally challenged with sources derived from scrapie.

Western blot analysis also revealed slight but consistent differences: the apparent molecular mass of the unglycosylated PrPres protein band in the brain of mice challenged with source derived from ovine BSE, as compared to that derived from bovine BSE by either intra-cerebral or oral route, was consistently lower (0.3–0.4 kDa) whereas the glycoform ratios were the same. These results are similar to those previously described between BSE in cattle and sheep [28], but here, surprisingly, they are observed after passage in a same experimental model expressing the ovine PrP protein. Such differences in PrPres molecular mass were not found in the mouse spleens. The reason why the passage history seems to result in a faint difference in PrPres molecular signature is unclear, and the extent to which this could be associated with possible changes in the pathobiological properties of the BSE agent remains unknown. Such changes have been shown to occur after passage in sheep [35], [36], [37]. It should also be noted that the attack rate from strain derived from ovine BSE was surprisingly lower and PrPres remained undetected in most (4/5) of the mouse spleens after oral challenge. In addition to possible changes after a passage in sheep, a possible reduction of the capacity of the source derived from ovine BSE agent to propagate by the oral route, following the first two passages that were performed by the intra-cerebral route in a transgenic mouse model characterized by a neuron-specific pattern of expression, cannot be totally excluded.

The previous sources derived from classical scrapie and BSE were the only TSEs examined that were transmissible by the oral route in TgOvPrP4 mice. No transmission by the oral route was achieved with other TSE sources originating from the experimental scrapie sources 87V and CH1641, two natural, “CH1641-like” or Nor98, scrapie isolates, an L-type BSE isolate, or from an isolate of transmissible mink encephalopathy (TME) passaged in cattle. H-type BSE and CWD were not examined as we had been unable to transmit these diseases by the intra-cerebral route in the TgOvPrP4 mouse model [18] (unpublished data). The titers of the inocula used in this study were not determined but transmission had been demonstrated unequivocally by the intra-cerebral route for all the inocula. These results might be partially explained by the pattern of tissue-specific PrP expression in TgOvPrP4 mice, under the control of the neuron-specific enolase promoter, as less neuroinvasive TSEs have been reported to require amplification in the follicular dendritic cells of the lymphoid tissues prior to neuroinvasion via the peripheral nerves [26], [38].

It is indeed apparent that the orally transmissible sources derived from classical BSE and scrapie were also able to propagate readily in the spleens of TgOvPrP4 mice, after both intra-cerebral and oral challenges. Among other sources that failed to transmit by the oral route, only L-BSE and bovine TME were lymphotropic by the intra-cerebral route at least during serial passages [18]. As regards the scrapie sources and their transmissibility by peripheral routes, failure to transmit 87V by the oral route had already been reported in sheep, whereas the ME7, 79A and 22A murine strains were transmissible [39]. Very low efficiency in transmitting this 87V strain was also reported in mice by intra-peritoneal route [40]. Also, concerning the ovine scrapie sources, failure to transmit CH1641 scrapie by subcutaneous inoculation was reported in sheep [41]. Oral transmission of Nor98 scrapie has been reported in sheep, but only of the A_136_ H_154_ Q_171_ homozygous *prnp* genotype [42], thus differing in this respect from TgOvPrP4 mice. The failure of both sources derived from L-BSE and bovine TME to be transmitted by the oral route in TgOvPrP4 is more intriguing. These are the most rapid ruminant TSEs in TgOvPrP4 mice by intra-cerebral route, which makes the results even more significant as transmission of the disease might be expected to occur within the life span of the mice, as observed for sources derived from classical BSE. Moreover we previously reported that L-BSE was readily transmissible from cattle by the oral route in another experimental model, the mouse lemur [43]. In the case of TME, our strain typing studies in several experimental models highlighted similarities with L-BSE and suggested that TME could be the result of a food borne transmission of L-BSE in ranch-raised minks [18], [44]. Transmission of these TSEs might involve particular pathways that could be affected in the TgOvPrP4 transgenic mouse model. Besides, our results could be influenced by the protocol used for experimental challenge involving intra-gastric administration, meaning that the inocula were not chewed by the mice, whereas in the case of experiments with L-BSE in lemurs, inocula were mixed to food. Alternatively, we can again consider the hypothesis that oral transmissibility could have been reduced by previous passages by the intra-cerebral route during the two passages in TgOvPrP4 mice, while initial brain samples of ruminants were not analyzed in this first oral route study.

However our results reveal the transmissibility of some TSEs by the oral route in a transgenic mouse model. Interestingly, some of our data suggest a possible influence of the passage history of inocula, including the inoculation routes or host features. These need careful consideration when interpreting data on the pathobiological properties of TSE agents in experimental models. To the best of our knowledge, this is the first report of oral transmission in a transgenic model expressing the prion protein of a species naturally affected by TSEs. It provides proof of principle that such models can constitute useful experimental tools for studies of oral transmission of prion diseases.

## Supporting Information

Table S1 **Overview of clinical signs observed in TgOvPrP4 ovine transgenic mice after transmission by the oral** ***versus*** **the intra-cerebral route.** The number of mice euthanized is indicated in each group. The possible presence of paresis, prostration or thinness is specified. ND: no data.
(DOCX)
