# Supplementary material for: Distinct Transmissibility Features of TSE Sources Derived from Ruminant Prion Diseases by the Oral Route in a Transgenic Mouse Model (TgOvPrP4) Overexpressing the Ovine Prion Protein
Source: PLoS One. 2014 May 5;9(5):e96215. doi: 10.1371/journal.pone.0096215 (PMC4010433; doi:10.1371/journal.pone.0096215)
Supplement: Table S1 — Overview of clinical signs observed in TgOvPrP4 ovine transgenic mice after transmission by the oral versus the intra-cerebral route. The number of mice euthanized is indicated in each group. The possible presence of paresis, prostration or thinness is specified. ND: no data. (DOCX) [file pone.0096215.s001.docx]

| TSE sources |  | Euthanized / inoculated | | | |  | Paresis | | | | | |  | Prostration | | | | | | |  | Thinness | | |
| --- | --- | --- | --- | --- | --- | --- | --- | --- | --- | --- | --- | --- | --- | --- | --- | --- | --- | --- | --- | --- | --- | --- | --- | --- |
|  |  | 2^nd^ i.c. | 3^rd^  i.c. | 3^rd^  oral | |  | 2^nd^ i.c. | 3^rd^  i.c. | 3^rd^  oral | | | |  | 2^nd^ i.c. | | | 3^rd^  i.c. | | | 3^rd^  oral |  | 2^nd^ i.c. | 3^rd^  i.c. | 3^rd^  oral |
|  |  |  |  |  | |  |  |  |  | | | |  |  | | |  | | |  |  |  |  |  |
| Natural ‘CH1641-like’ scrapie isolate |  | 9/12 | 10/11 | 5/6 | |  | **8/9** | **7/10** | 1/5 | | | |  | **5/9** | | | **10/10** | | | 2/5 |  | **6/9** | **5/10** | **4/5** |
| Experimental CH1641 scrapie isolate |  | 11/12 | 10/12 | 3/6 | |  | **11/11** | **9/10** | 0/3 | | | |  | **11/11** | | | **10/10** | | | 0/3 |  | 4/11 | **8/10** | **3/3** |
| Natural Nor98 scrapie isolate |  | 4/12 | ND | 5/6 | |  | **4/4** | ND | 1/5 | | | |  | **4/4** | | | ND | | | **4/5** |  | **3/4** | ND | **5/5** |
| Natural L-type BSE |  | 9/11 | 9/12 | 5/6 | |  | **9/9** | **8/9** | 2/5 | | | |  | **5/9** | | | **9/9** | | | 1/5 |  | **9/9** | **9/9** | **5/5** |
| Experimental bovine TME |  | 9/11 | 2/11 | 5/6 | |  | **9/9** | **2/2** | 0/5 | | | |  | **5/9** | | | **2/2** | | | **3/5** |  | **9/9** | **2/2** | **5/5** |
|  |  |  |  |  | |  |  |  |  | | | |  |  | | |  | | |  |  |  |  |  |
| Experimental scrapie strain 87V |  | 10/11 | 5/6 | 4/6 | |  | **9/10** | **3/5** | 1/4 | | | |  | **8/10** | | | **4/5** | | | **3/4** |  | 4/10 | **5/5** | **2/4** |
| Experimental scrapie strain C506m3 |  | 11/12 | 6/6 | 4/6 | |  | **10/11** | **6/6** | **3/4** | | | |  | **8/11** | | | **6/6** | | | **3/4** |  | 3/11 | 0/6 | **3/4** |
| Natural classical scrapie isolate |  | 8/11 | ND | 4/6 | |  | **7/8** | ND | **3/4** | | | |  | **8/8** | | | ND | | | **4/4** |  | 5/8 | ND | **2/4** |
|  |  |  |  |  | |  |  |  |  | | | |  |  | | |  | | |  |  |  |  |  |
| Experimental ovine C-type BSE |  | 12/12 | 11/12 | 4/6 | |  | **8/12** | **9/11** | 1/4 | | | |  | **8/12** | | | **8/11** | | | 1/4 |  | 1/12 | 3/11 | **4/4** |
| Natural C-type BSE |  | 10/11 | 7/11 | 3/6 | |  | **9/10** | **7/7** | 0/3 | | | |  | **6/10** | | | **7/7** | | | 0/3 |  | 4/10 | 2/7 | **2/3** |
|  |  |  | | |  |  | | | |  |  |  | | |  |  | |  |  |  |  |  |  |  |
